# Various Parts of *Helianthus annuus* Plants as New Sources of Antimalarial Drugs

**DOI:** 10.1155/2019/7390385

**Published:** 2019-11-26

**Authors:** Wiwied Ekasari, Dwi Widya Pratiwi, Zelmira Amanda, Suciati, Aty Widyawaruyanti, Heny Arwati

**Affiliations:** ^1^Department of Pharmacognosy and Phytochemistry, Faculty of Pharmacy, Universitas Airlangga, Surabaya 60115, Indonesia; ^2^Department of Medical Parasitology, Faculty of Medicine, Universitas Airlangga, Surabaya 60115, Indonesia

## Abstract

**Background:**

Each part of *H. annuus* plants is traditionally used as medicinal remedies for several diseases, including malaria. Antimalarial activity of the leaf and the seed has already been observed; however, there is no report about antimalarial activity of the other parts of *H. annuus* plants. In this study, we assess *in vitro* and *in vivo* antimalarial activity of each part of the plants and its mechanism as antimalarial agent against inhibition of heme detoxification.

**Objective:**

To investigate the antimalarial activity of various parts of *H. annuus*.

**Methods:**

Various parts of the *H. annuus* plant were tested for *in vitro* antimalarial activity against *Plasmodium falciparum* 3D7 strain (chloroquine-sensitive), *in vivo* antimalarial activity against *P. berghei* using Peters' 4-day suppressive test in BALB/c mice, curative and prophylaxis assay, and inhibition of heme detoxification by evaluating *β*-hematin level.

**Results:**

Ethanol extract of the roots showed the highest antimalarial activity, followed by ethanol extract of leaves, with IC_50_ values of 2.3 ± 1.4 and 4.3 ± 2.2 *μ*g/mL, respectively and the percentage inhibition of *P. berghei* of 63.6 ± 8.0 and 59.3 ± 13.2 at a dose of 100 mg/kg, respectively. Ethanol extract of roots produced an ED_50_ value of 10.6 ± 0.2 mg/kg in the curative test and showed an inhibition of 79.2% at a dose of 400 mg/kg in the prophylactic assay. In inhibition of heme detoxification assay, root and leaf ethanol extracts yielded a lower IC_50_ value than positive (chloroquine) control with a value of 0.4 ± 0.0 and 0.5 ± 0.0 mg/mL, respectively.

**Conclusion:**

There were promising results of the ethanol extracts of root of *H. annuus* as a new source for the development of a new plant-based antimalarial agent.

## 1. Introduction

Most developing countries, including Indonesia, still depend on using plants in traditional medicine, including antimalarial agents. One of these plants is *Helianthus annuus* (Asteraceae), known as sunflower or “*bunga matahari*” in local language. Traditionally, each part of *H. annuus* plants is widely used in several healthcare applications, including the treatment of malaria. Sunflower tea is reported to be useful in the treatment of malarial and lung disease [1], and the leaves have long been used in infusion and decoction as traditional remedy for malaria by Ambalabe village community, Madagascar [2].

Muti'ah et al. [3] report that 80% ethanol extract of *H. annuus* leaf shows good antimalarial activity with an ED_50_ value of 4.64 mg/kg. Moreover, the combination of 80% ethanol extract of *H. annuus* leaf and *Acalypha indica* Linn. leads to synergetic effect in *in vivo* antimalarial assay with a CI value of 0.46 (CI < 1) and an ED_50_ value of 1.23 mg/kg [4]. Intisar et al. [5] also report that methanol and petroleum ether extract from *H. annuus* seeds showed good inhibition activity against *P. falciparum* strain KI with an EC_50_ of 0.19 and 0.34 *μ*g/mL, respectively.

Based on the data above, it is recognized that *H. annuus* plants are potential source of antimalarial drugs. However, antimalarial activity of the other parts of *H. annuus* plants has not been reported to date. Thus, in this study, we assess *in vitro* and *in vivo* antimalarial activity of each part of the plant and its mechanism as antimalarial agent against the inhibition of heme detoxification. The inhibition of heme detoxification leads to growth disorder and malaria parasites' mortality as a result of membrane lysis and activity disorder of some enzymes [6–9]. Hence, a substance with high heme detoxification inhibition activity is considered a potential antimalarial agent. Inhibition of heme detoxification is one of the potential biochemical targets, which is possessed by chloroquine and artemisinin [10, 11]. The highest antimalarial activity of *H. annuus* plant parts and its ability to inhibit heme detoxification are expected to be obtained through this study as it can be a new source of antimalarial drugs.

## 2. Materials and Methods

### 2.1. Plant Materials

*H. annuus* plants were obtained from Oro-oro Ombo, Malang, East Java, Indonesia, in November 2016, and determined by Materia Medica Batu, Malang, East Java. Each part of the plant, including roots, stems, seeds, flowers, and leaves, were air-dried separately. Samples were then ground to powder, once they are completely dried.

### 2.2. Animal Model

Adult male BALB/c mice with 20–30 g of bodyweight were obtained from Faculty of Veterinary Medicine, Universitas Airlangga. The mice were fed with mice pellet diet and given free access to drink clean water. The animals were allowed to acclimatize for two weeks before being treated. The permission and approval for animal studies were obtained from Faculty of Veterinary Medicine, Universitas Airlangga.

### 2.3. Preparation of 96% Ethanol Extract of *H. annuus*

Powders from each part of *H. annuus* plants (50 gram) were macerated by 96% ethanol and then evaporated on a rotary evaporator.

### 2.4. *In Vitro* Antimalarial Assay

In this research, cultures of *Plasmodium falciparum* strain 3D7 (chloroquine-sensitive) were used and cultivated using the Trager and Jensen method [12]. Cultures were cultivated in human O^+^ red blood cells with 5% hematocrit in RPMI 1640 (Gibco BRL, USA) added with 22.3 mM HEPES (Sigma), hypoxanthine, sodium bicarbonate, and 10% human O^+^ plasma. Chloroquine diphosphate was used as the positive control.

Antimalarial assay was done using a 24-well microplate with 1% initial and experimental parasitaemia (1 mL/well of suspension), followed by incubation in a multigas incubator at 37°C for 48 hours. The incubated materials were then collected and used to make a thin smear in a glass slide, fixated in methanol, and stained with Giemsa. The number of parasites was then observed under a microscope and compared with the negative control to determine the percentage of parasite growth inhibition. IC_50_ value was calculated using Probit Analysis.

### 2.5. *In Vivo* Antimalarial Activity Assay

*Plasmodium berghei* strain ANKA was obtained from Eijkman Institute of Molecular Biology, Jakarta, Indonesia. About 20% of parasitaemia-infected mice were dissected and then the intracardial blood was taken and diluted with the PBS. Each mouse used in this research was inoculated priorly intraperitoneally with 0.2 mL of solution containing 1 × 10^7^*P. berghei*-infected erythrocytes [13].

*In vivo* antimalarial assay of the plant extract was done using Peter's method [14], which had been modified with 100 mg/kg/day as the selected dose. Thirty-six adult male BALB/c mice (25 g ± 3) were inoculated with parasites and randomly grouped into six groups (five treatment groups and one control group), with six mice in each group. Negative control group was treated with 0.5% sodium carboxymethyl cellulose (Na CMC) suspension. Treatment groups were treated with extract suspension of each part of the plant with the selected dose. Suspension was given orally once a day for 4 days. On the fourth day, a thin blood smear was made from the tail of each mouse, fixated in methanol, and then stained with Giemsa. The thin blood smears were then observed under a microscope to calculate the percentage of parasitaemia and parasite inhibition.

### 2.6. *In Vivo* Curative Assay of 96% Ethanol Extract of *H. annuus* Roots

Thirty adult male BALB/c mice (8 weeks old) were randomly divided into five groups (six mice per group). Each mouse was infected intraperitoneally with 0.2 mL of solution containing 1 × 10^7^*P. berghei*-infected erythrocytes. Negative control (group 1) was treated with 0.5% Na CMC suspension. Groups 2, 3, 4, and 5 were treated with 96% ethanol extract suspension of *H. annuus* root with 1, 10, 100, and 250 mg/kg dose per mouse. Suspension was given orally once a day for 4 days. Percentage of parasitaemia was calculated by making a thin blood smear on the fourth day, which is then fixated in methanol and stained with Giemsa. The calculation was then compared with the control group. ED_50_ value was obtained by using Probit Analysis.

### 2.7. *In Vivo* Prophylaxis Assay of 96% Ethanol Extract of *H. annuus* Roots

Thirty adult male BALB/c mice (8 weeks old) were randomly grouped into six groups (five mice per group). Groups 1, 2, 3, and 4 were treated with 96% ethanol extract suspension of *H. annuus* root with 100, 200, 400, and 800 mg/kg dose per mouse. Group 5 as the negative control group was treated with 0.5% Na CMC suspension, while the positive control (group 6) was given 13 mg/kg of doxycycline suspension [15]. Each suspension was given orally once a day for 4 days. On the fourth day, each mouse was inoculated with 0.2 mL of solution containing 1 × 10^7^*P. berghei*-infected erythrocytes by intraperitoneal injection. Percentage of parasitaemia was determined on the third day by observing the thin blood smear and compared with the control group. Mean survival time of mice in each group was determined during 14 days (D_0_–D_13_). Mean survival time was calculated using the following equation:

(1) $$\begin{array}{c} \text{mean survival time}\left(\text{MST}\right)=\frac{\text{sum of survival days}}{\text{total observation days}\left(14\right)}\times 100. \end{array}$$

### 2.8. Heme Detoxification Inhibition Assay

Inhibition of heme detoxification was assessed using the Basilico method [16] with modification. The extract and positive control (chloroquine diphosphate) were diluted with dimethyl sulfoxide (DMSO) a concentration range of 2–0.01 mg/mL. The amount of hematin present in each sample was calculated using a standard curve of hematin dissolved in 0.2 M NaOH. The absorbance was read using the ELISA reader at a wavelength of 405 nm. The effect of each sample against the production of *β*-hematin was calculated and compared with the negative control [17, 18].

### 2.9. Statistical Analysis

Data are expressed as mean ± standard deviation (SD). IC_50_ values were calculated using Probit Analysis. Statistical significance was determined by Kruskal–Wallis nonparametric independent *t*-test and ANOVA (*one-way*).

## 3. Results and Discussion

*H. annuus* has been planted widely in Indonesia and long been used traditionally as anti-inflammatory, antimalarial, antiasthma, antioxidant, antitumor, and antimicrobial agent. It has been reported that several chemical compounds were already isolated and identified from various parts of this plant, such as heliangolides (sesquiterpene lactones), *α*-pinene, sabinene (monoterpene), helikauranoside (diterpene), and alkaloid and phenolic group in the leaf part; helianthoside (triterpene) and saponin group in the flower part; fatty acid, tocopherol, tannin, and polyphenol group in the seed part; alkaloid and phenolic group in the stem and root parts [1]. Extraction yield from each part of *H. annuus* plants macerated with 96% ethanol is shown in Table 1.

*In vitro* screening to observe *P. falciparum* inhibition activity of the plant extract with IC_50_ value less than 10 *μ*g/mL is an important primary step in the development of new antimalarial drugs. Plant extracts showing IC_50_ values ≤10 *μ*g/mL can be considered to be active, IC_50_ values in the range 10 < IC_50_ ≤ 25 *μ*g/mL can be considered to be moderately active, and those exhibiting IC_50_ values >25 *μ*g/mL can be considered to be inactive [19]. IC_50_ values of 96% ethanol extract of various *H. annuus* plant parts are shown in Table 2. Root extract exhibits the highest *in vitro* antimalarial activity, followed by leaf and flower extracts with an IC_50_ value of 2.3 ± 1.4, 4.3 ± 2.2, and 4.8 ± 0.0 *μ*g/mL, respectively.

*In vivo* antimalarial activity was also determined for each extract from various parts of *H. annuus* plants, and the results are shown in Table 3. Ethanol extract of root exhibits the highest percentage inhibition against *P. berghei*, followed by leaf extract with the percentage inhibition of 63.6 ± 8.0 and 59.3 ± 13.2, respectively.

Method and criteria are varied among the treatment groups examining antimalarial potency of plants by using the rodent animal model. *P. berghei*-infected mice given orally 50–250 mg/kg/day of extract exhibiting inhibition percentage >60% are considered to be active or very active, and those exhibiting inhibition percentage >30% are considered to be moderately active [20–24]. In this research, oral administration at a dose of 100 mg/kg/day of ethanol *H. annuus* extract is used as an initial experiment and observation of parasite growth up to day 4 using mice model.

Based on the first screening of *in vivo* assay, it is shown that *H. annuus* root extract has the highest activity. Thus, the experiment is continued to assess curative and prophylaxis antimalarial activity of the ethanol root extract of *H. annuus*.

In *in vivo* curative assay, *P. berghei*-infected mice are treated orally with 96% ethanol root extract at a dose of 1, 10, 100, and 250 mg/kg. The results of the 4-day suppressive test show that the ethanol extract of *H. annuus* root has an ED_50_ value of 10.6 ± 0.2 mg/kg (Table 4). According to Munoz et al. [25], antimalarial activity can be categorized based on the ED_50_ value, where ED_50_ <100 mg/kg is considered to have very good activity.

**Figure 1:**
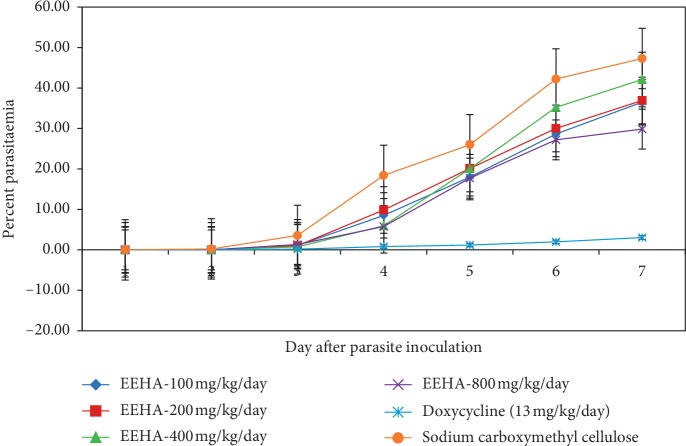
Percentage of parasite growth during seven days after inoculation with parasites on *in vivo* prophylaxis assay of 96% ethanol extract of *H. annuus* roots. Average values and standard deviations from five mice per drug/dose are also shown. *p* value <0.05. EEHA = ethanolic extract of *H. annuus*.

In prophylactic effect evaluation of 96% ethanol *H. annuus* root extract, mice are given a sample orally for 4 days before inoculating with the parasite. The results (see Figure 1) show that the percentage of inhibition parasitaemia in mice treated with the extract at a dose of 400 mg/kg is higher than that in those treated with 800 mg/kg. Despite that, this result is still lower than that of doxycycline, a standard drug for prophylactic antimalarial in Indonesia.

The 400 mg/kg dose group shows the highest percentage of prophylactic inhibition (79.2%) at day 3, and it declines to 62.5% at a dose of 800 mg/kg. Calabrese and Baldwin [26] report that the upside-down U-curve in the drug activity assessment at different doses may indicate the occurrence of the hormetic effect, which points out to the toxicity of the drug. Aside from the declining percentage of inhibition, the low mean survival time at a dose of 800 mg/kg, compared to 200 and 400 mg/kg doses, indicates that the *H. annuus* root extract at high dose (800 mg/kg) tends to have toxicity.

The results of Kruskal–Wallis nonparametric independent *t*-test and *one-way* ANOVA analysis show that there are significant differences (*p* value < 0.05) in inhibition percentage of the positive control group compared with all the sample groups and the negative control group. There is no significant difference between 100, 200, 400, and 800 mg/kg doses.

Figure 1 shows that, 96 hours or the fourth day after parasite inoculation, there is an increase in the parasitic growth in the negative dose and control group, whereas in the doxycycline group the parasite growth is relatively stable, which is below 3%. This is thought to be related to the half-life of doxycycline which ranges from 12 to 22 hours [15, 27, 28] allowing doxycycline to last longer in the body and inhibit the growth of parasites. It is in contrast to extracts or traditional medicines that tend to have a short half-life, such as artemisinin and its derivatives [29, 30]. Based on the antimalarial test results of *H. annuus* root *in vivo*, it can be concluded that the 96% ethanol extract of *H. annuus* root has more potential activities as curative antimalarial than prophylactic.

One of the potential biochemical targets for the development of new antimalarial drugs is heme detoxification. The results of heme detoxification inhibition assay (see Table 5) show that the 96% ethanol extract of *H. annuus* root (IC_50_ = 0.4 ± 0.0 mg/mL) and leaf (IC_50_ = 0.5 ± 0.0 mg/mL) has higher activity than chloroquine diphosphate, a standard antimalarial drug (IC_50_ = 0.6 ± 0.0 mg/mL).

It was reported that free heme is toxic to parasites as it causes lysis of the parasite vacuolar membrane [31]. Thus, malaria parasites develop heme detoxification mechanism to change the toxic heme into a harmless one. The inhibition of heme detoxification leads to growth disorder and malaria parasites mortality as a result of membrane lysis and activity disorder of some enzymes [6–9].

Based on the entire assay undertaken in this research, it is established that the ethanol extract of *H. annuus* root shows the highest antimalarial activity among the other parts of plants, either in *in vitro*, *in vivo*, or in heme detoxification inhibition assay. Therefore, the ethanol extract of *H. annuus* root is a potential source of new antimalarial drugs from natural resources.

## 4. Conclusion

Based on the results of the study, they show that 96% ethanol extract of *H. annuus* root has the highest antimalarial activity compared to stems, seeds, flowers, and leaves of *H. annuus*. 96% ethanol extract of *H. annuus* root has more potential activity as a curative antimalarial drug than a prophylactic one. Similarly, for the antimalarial mechanism in inhibiting heme detoxification, 96% ethanol extract of *H. annuus* root has the greatest inhibitory activity compared to the other parts of *H. annuus*; even the inhibition is greater than chloroquine, a standard drug. So, it can be concluded that the 96% ethanol extract of *H. annuus* root contains a potential antimalarial substance which is a new source for the development of a new plant-based antimalarial agent.

## Figures and Tables

**Table 1:** Extraction yield from various parts of *H. annuus* plants.

*H. annuus* plant parts	Extraction yield (%)
Roots	2.1
Stems	5.0
Seeds	2.9
Flowers	6.7
Leaves	4.4

**Table 2:** IC_50_ values of 96% ethanol extract of various parts of *H. annuus* plants against *P. falciparum* strain 3D7.

*H. annuus* plant parts	% inhibition at each concentration (*μ*g/mL)					IC_50_ (*μ*g/mL)
	100	10	1	0.1	0.01	
Roots	86.6 ± 1.7	73.3 ± 6.9	32.0 ± 3.7	22.3 ± 0.0	7.3 ± 7.4	2.3 ± 1.4
Stems	64.4 ± 4.6	56.4 ± 1.1	30.7 ± 7.7	18.8 ± 4.9	0	10.2 ± 5.0
Seeds	84.4 ± 1.0	22.2 ± 6.9	14.2 ± 3.9	6.2 ± 10.3	0	19.3 ± 5.5
Flowers	85.7 ± 8.7	48.4 ± 2.6	32.0 ± 2.9	19.3 ± 8.7	0	4.8 ± 0.0
Leaves	96.3 ± 1.8	47.1 ± 4.7	24.3 ± 11.7	16.5 ± 6.2	7.0 ± 1.0	4.3 ± 2.2

**Table 3:** Mean percentage of parasitaemia, growth, and inhibition in *P. berghei*-infected mice treated with 96% ethanol *H. annuus* extract at a dose of 100 mg/kg orally once a day for 4 days.

*H. annuus* plant parts	Mean % parasitaemia		Mean % growth	Mean % inhibition
	Day 0	Day 4		
Roots	0.9 ± 0.2	4.6 ± 1.0	3.7 ± 0.8	63.6 ± 8.0
Stems	1.2 ± 0.2	7.3 ± 1.0	6.1 ± 0.8	40.3 ± 8.2
Seeds	1.0 ± 0.2	7.1 ± 0.5	6.0 ± 0.4	41.5 ± 4.4
Flowers	1.2 ± 0.5	7.8 ± 0.4	6.6 ± 0.5	35.9 ± 4.6
Leaves	0.9 ± 0.2	5.1 ± 1.5	4.2 ± 1.4	59.3 ± 13.2
Na CMC	1.6 ± 0.3	11.8 ± 0.9	10.3 ± 0.9	—

**Table 4:** Mean percentage of parasitaemia, growth, and inhibition against *P. berghei* of 96% ethanol *H. annuus* root extract compared with control.

Sample	Dose (mg/kg)	Mean % parasitaemia		Mean % growth	Mean % inhibition	ED_50_ (mg/kg)
		D_0_	D_4_			
96% ethanol extract of *H. annuus* root	1	1.1 ± 0.1	26.2 ± 1.4	6.3 ± 0.4	35.8 ± 4.7	10.6 ± 0.2
	10	1.0 ± 0.2	21.2 ± 0.9	5.1 ± 0.2	48.3 ± 2.8	
	100	1.0 ± 0.1	15.4 ± 1.7	3.6 ± 0.4	64.0 ± 5.3	
	250	1.2 ± 0.4	12.0 ± 1.8	2.7 ± 0.4	72.3 ± 5.3	
Na CMC	—	1.2 ± 0.3	40.3 ± 6.1	9.8 ± 1.5	—	

**Table 5:** Results of heme detoxification inhibition assay of the 96% ethanol extract of each part of the *H. annuus* plant.

Sample	Concentration (mg/mL)	Mean value of hematin (mM)	Mean % inhibition	IC_50_ (mg/mL)
Negative control	—	121.0 ± 2.6	—	—
96% ethanol extract of root	2.00	34.2 ± 1.2	71.7 ± 1.0	0.4 ± 0.0
	1.00	44.6 ± 1.2	63.1 ± 1.0	
	0.50	58.3 ± 1.1	51.8 ± 0.9	
	0.25	67.6 ± 1.3	44.1 ± 1.1	
	0.10	81.8 ± 1.3	32.3 ± 1.1	
	0.01	99.8 ± 2.2	17.5 ± 1.8	

96% ethanol extract of seed	2.00	61.6 ± 1.4	49.1 ± 1.1	2.4 ± 0.2
	1.00	71.0 ± 1.2	41.3 ± 1.0	
	0.50	84.9 ± 3.3	29.8 ± 2.8	
	0.25	97.3 ± 2.3	19.6 ± 1.9	
	0.10	105.5 ± 1.3	12.8 ± 1.1	
	0.01	114.1 ± 1.8	5.7 ± 1.5	

96% ethanol extract of flower	2.00	46.8 ± 1.1	61.4 ± 0.9	0.9 ± 0.1
	1.00	55.7 ± 1.3	53.9 ± 1.1	
	0.50	68.4 ± 0.9	43.4 ± 0.7	
	0.25	81.8 ± 0.9	32.4 ± 0.7	
	0.10	89.8 ± 0.9	25.8 ± 0.8	
	0.01	99.3 ± 1.2	17.9 ± 1.0	

96% ethanol extract of leaf	2.00	38.6 ± 0.9	68.1 ± 0.7	0.5 ± 0.0
	1.00	48.9 ± 0.8	59.6 ± 0.7	
	0.50	62.0 ± 1.1	48.7 ± 0.9	
	0.25	75.5 ± 1.4	37.6 ± 1.1	
	0.10	83.5 ± 0.6	31.0 ± 0.5	
	0.01	98.1 ± 0.9	18.9 ± 0.7	

96% ethanol extract of stem	2.00	140.1 ± 9.2	53.7 ± 3.1	1.9 ± 0.5
	1.00	179.8 ± 5.6	40.6 ± 1.8	
	0.50	206.3 ± 8.3	31.8 ± 2.8	
	0.25	241.8 ± 3.6	20.1 ± 1.2	
	0.10	264.7 ± 3.6	12.5 ± 1.2	
	0.01	288.0 ± 5.5	4.8 ± 1.8	

Chloroquine diphosphate	2.00	40.4 ± 2.1	66.6 ± 1.7	0.6 ± 0.0
	1.00	50.9 ± 0.6	57.9 ± 0.5	
	0.50	64.3 ± 0.9	46.8 ± 0.7	
	0.25	77.0 ± 0.8	36.3 ± 0.7	
	0.10	85.3 ± 1.2	29.4 ± 1.0	
	0.01	94.6 ± 0.7	21.8 ± 0.6	

## Data Availability

All data are included within the manuscript.
